# Recent Advance in Electrochemical Chiral Recognition Based on Biomaterials (2019–2024)

**DOI:** 10.3390/molecules30163386

**Published:** 2025-08-14

**Authors:** Shan Qiu, Guo-Ying Chen, Yi-Dan Qin, Ting-Ting Li, Feng-Qing Yang

**Affiliations:** School of Chemistry and Chemical Engineering, Chongqing University, Chongqing 401331, China; 202418021112T@stu.cqu.edu.cn (S.Q.); 20221801017@stu.cqu.edu.cn (G.-Y.C.); 202418021097T@stu.cqu.edu.cn (Y.-D.Q.); 202418021033@stu.cqu.edu.cn (T.-T.L.)

**Keywords:** chiral recognition, electrochemical chiral sensors, chiral selectors, biomaterials

## Abstract

Chirality is a prevalent characteristic of natural systems that plays a significant role in the biological activities of living organisms, and the enantiomers typically exhibit different pharmacological activities. Consequently, developing methods with high selectivity and sensitivity for chiral analysis is of great importance for pharmaceutical engineering, biomedicine, and food safety. Electrochemical chiral recognition has garnered significant attention owing to its unique advantages, including simplicity of operation, rapid response, and cost-effectiveness. The biomaterials, such as amino acids, proteins, nucleic acids, and polysaccharides, possess inherent chiral sites, excellent biocompatibility, and abundant modifiable groups, rendering them ideal candidates for constructing electrochemical chiral sensors. This review focuses on the research progress of electrochemical chiral recognition based on different biomaterials from 2019 to 2024. In addition, the distinct chiral recognition mechanisms and electrochemical analysis methods, as well as the research challenges and prospects of electrochemical chiral sensors based on biomaterials in enantiomer recognition are discussed. This review can provide a reference for further study in related fields.

## 1. Introduction

Chirality is a fundamental attribute of biological systems, referring to the characteristic that an object cannot be perfectly superimposed on its mirror image, which is analogous to the distinction between a person’s left and right hands. Enantiomers are commonly present in pesticides [1], pharmaceuticals [2], and food [3]. In non-chiral environments, they generally exhibit similar physical and chemical properties; nevertheless, in chiral environments, they demonstrate distinct or even opposite effects in pharmacology, toxicology, and metabolic pathways [4,5,6]. For example, the R enantiomer of thalidomide [7] exhibits sedative effects that can effectively alleviate pregnancy-related symptoms in pregnant women, while the S enantiomer possesses a significant teratogenic property. In the 1960s, the insufficient comprehension of chiral medicines resulted in a large number of fetal malformations caused by thalidomide. This tragic event swiftly drew attention to research on chiral separation techniques, enantiomer-selective analysis methods, and the development of single-enantiomer drugs.

Numerous techniques have been reported for chiral recognition, including high-performance liquid chromatography [8], gas chromatography [9], capillary electrophoresis [10], chemiluminescence spectroscopy [11], fluorescence spectroscopy [12], and circular dichroism spectroscopy [13], among others. However, these analytical methods are usually hindered by some disadvantages, including expensive instrumentation, time-consuming sample pretreatment, and complex procedures, which considerably restrict their applicability. Therefore, developing rapid, simple, low-cost, and highly sensitive techniques for chiral recognition and separation has emerged as an important research direction in analytical chemistry. In reality, electrochemical chiral recognition methods offer several advantages, such as simple operation, low cost, fast response, real-time detection, and ease of miniaturization, which have attracted great interest from scientific researchers [14,15]. Electrochemical chiral recognition is achieved through the selective binding of chiral host and guest molecules, resulting in different Gibbs free energy that are further converted to measurable electrochemical signals (current, voltage, impedance, etc.). The primary challenge of electrochemical chiral sensors lies in the construction of an efficient electrochemical sensing interface that can simultaneously satisfy the requirements of sufficient chiral recognition sites and an excellent electrochemical signal transduction capability.

It should be noted that traditional electrodes cannot directly perform electrochemical chiral recognition. Typically, chiral recognition materials [16,17,18] containing chiral selectors need to be modified on the electrode surface to provide chiral recognition sites. In recent years, biomaterials have been extensively studied in the field of chiral separation science, encompassing amino acids and their derivatives [19], polysaccharides and their derivatives [20], proteins [21], enzymes [22], and nucleic acids [23]. Besides possessing outstanding biocompatibility, biomaterials have intrinsic chiral structures and precise molecular recognition sites, enabling the specific recognition of enantiomers through stereoselective interactions. Their abundant functional groups (such as amino, hydroxyl, carboxyl, and thiol groups) provide key reaction sites for chemical modifications, functional regulations, and intermolecular interactions. Hence, biomaterials are often employed as chiral selectors to construct electrochemical chiral sensors. In addition, the biomaterials need to be integrated with other conductive materials to amplify the electrochemical response signal, thereby establishing a stable and highly selective chiral sensing interface.

**Figure 1:**
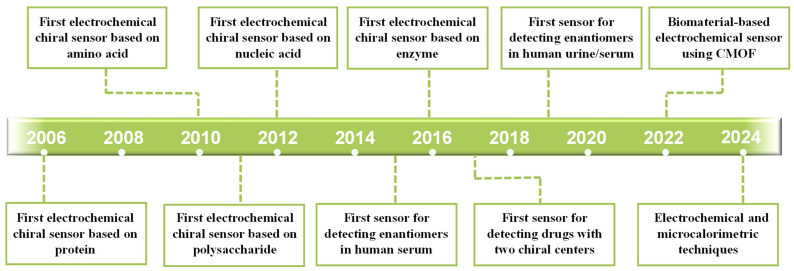
Timeline summary of important events in the development of biomaterial-based electrochemical chiral sensors.

Figure 1 shows ten significant contributions that have advanced the development of electrochemical chiral sensors based on biomaterials. The earliest work in this field was described in 2006; Wang et al. [24] developed a chiral sensor based on bovine serum albumin (BSA) for the detection of tryptophan enantiomers. Since then, various biomaterials have been applied in electrochemical chiral sensing, such as the first electrochemical chiral sensor for detecting tryptophan enantiomers using amino acid as the chiral selector in 2010 [25], the first sensor based on polysaccharides for detecting tryptophan enantiomers in 2011 [26], the first nucleic acid-based sensor for identifying zinc-fingers like metallo-supramolecular enantiomers in 2012 [27], the first real sample application (human serum) in 2015 [28], the first enzyme-based senor for L-amino acid detection (L-tryptophan, L-phenylalanine, and L-tyrosine) in 2016 [29], the first biomaterial-based sensor for detecting two-chiral-center drugs (moxifloxacin hydrochloride) in 2017 [30], the successful application on a real sample of human urine and serum in 2019 [31], identification of tryptophan enantiomers using a chiral metal–organic framework (D-His-ZIF-8)-modified sensor in 2022 [32], and electrochemical and microcalorimetric techniques for elucidating the interaction between chiral selectors and enantiomers in 2024 [33], among a large number of other examples.

This review provides an overview of the research advancement in biomaterial-based electrochemical chiral sensors (Table 1) for enantiomer recognition from 2019 to 2024. The mechanisms of electrochemical chiral recognition are summarized, the application characteristics of different electrochemical detection techniques are compared, and the key challenges and prospects in the field of electrochemical chiral recognition are discussed.

## 2. Mechanisms and Methods of Electrochemical Chiral Recognition

### 2.1. Mechanisms of Electrochemical Chiral Recognition

Previous studies indicate that chemical recognition and signal transduction are the two primary processes involved in chiral recognition. The paramount element of electrochemical chiral recognition is the interaction between the chiral selector and the enantiomers, resulting in the production of diastereomeric complexes with distinct solvation or formation equilibrium constants [81]. The varying Gibbs free energy of the diastereomeric complexes generated between the two enantiomers and the chiral selector is transformed to detectable electrical signals for chiral recognition. Ogston [82] initially employed the three-point interaction model in 1948 to elucidate the recognition process of chiral enzymatic reactions, which was further refined by other scientists [83,84,85]. The three-point interaction model involves a minimum of three interaction sites between the chiral selector and the enantiomers, with either attractive or repulsive interactions. Moreover, the intensity of these forces depends on the stereochemical structure of the molecules during the recognition process [86], with one of them varying due to the stereochemistry. The differentiation of three contact forces is crucial to correctly identify the enantiomers by chiral selector. The interaction forces comprise Coulomb forces, hydrogen bonds, π–π interactions, electrostatic effects, steric hindrance, and van der Waals forces [87]. The most potent interaction is the Coulomb force, resulting from the attraction or repulsion of electric charges. The strength of hydrogen bonds is considerable, owing to atoms with higher electronegativity approaching the hydrogen atom, thereby reducing the electron repulsion force [81]. Atoms or functional groups occupy a certain amount of space, resulting in steric hindrance that exhibits strong repulsive properties at a short distance [88]. Consequently, electrochemical chiral recognition relies on multiple forces between the selector and enantiomers. Chiral selectors can create diastereomeric complexes with enantiomers via these interactions, which exhibit distinct binding constants and varying Gibbs free energy, thus successfully differentiating the enantiomers. Based on the three-point interaction model, the types of chiral recognition can be mainly divided into chiral ligand exchange recognition, host–guest recognition, molecular imprinting recognition, and biological macromolecule recognition.

**Figure 2:**
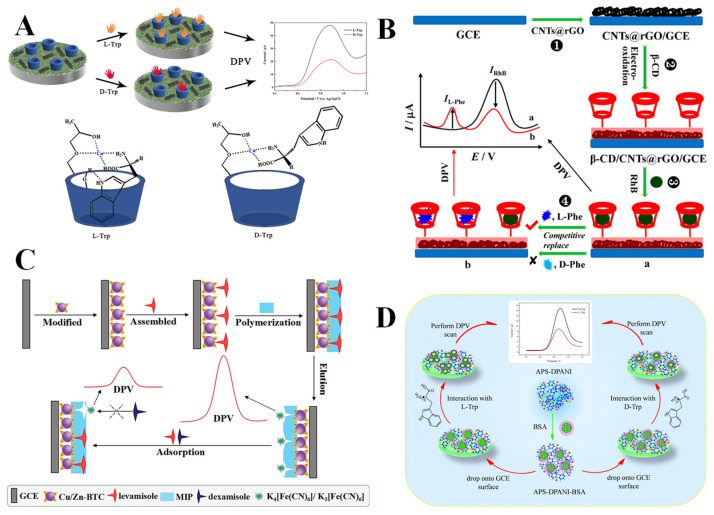
(**A**) Chiral recognition of Trp enantiomers based on chiral ligand exchange [57]. (**B**) Chiral recognition of Phe enantiomers based on host–guest recognition [58]. (**C**) Chiral recognition of levamisole based on molecular imprinting technology [90]. (**D**) Chiral recognition of Trp enantiomers based on biological macromolecule recognition [70].

Chiral ligand exchange recognition involves the ligand exchange between a chiral metal complex and an enantiomer, resulting in the formation of a diastereomeric ternary complex consisting of a metal ion, a chiral ligand, and an enantiomer. The spatial structural differences among various enantiomers result in diastereomeric ternary complexes with distinct structural stabilities and Gibbs free energy, which can be manifested in an electrochemical signal, facilitating the effective recognition of enantiomers. In electrochemical chiral ligand exchange recognition, the predominant utilized metal ions and chiral ligands are Cu^2+^ and cyclodextrins, along with their derivatives. Based on multi-walled carbon nanotubes (MWCNTs), hydroxypropyl-β-cyclodextrin (HP-β-CD), and carboxymethyl cellulose (CMC), Ji et al. [57] reported an efficient electrochemical sensor (GCE/MWCNTs/CMC-CD-Cu) for the chiral recognition of tryptophan enantiomers (L/D-Trp). Figure 2A illustrates that HP-β-CD can establish a chiral selective site through hydroxyl coordination to Cu^2+^, while its three-dimensional cavity provides a hydrophobic binding environment for the indole moiety of tryptophan. The variations in spatial conformation between L-Trp and D-Trp result in their different binding abilities to the Cu^2+^-HP-β-CD complex. The L-Trp is more readily accommodated within the HP-β-CD cavity, forming a stable ternary complex with the hydroxyl group at the outer edge of the cyclodextrin through hydrogen bonding. In contrast, the binding stability of D-Trp is markedly diminished due to the challenge in effectively overcoming spatial site resistance. Therefore, L-Trp displays a stronger binding affinity to the chiral sensing interface, demonstrating enhanced electron transfer efficiency and a more prominent electrochemical signal as compared with D-Trp, with low limits of detections (LODs) of 0.81 µM and 1.9 µM for L-Trp and D-Trp, respectively.

Host–guest recognition is a core mechanism in supramolecular chemistry, denoting the ability of a host molecule with a defined structure or cavity to selectively bind guest molecules and form host–guest inclusion complexes with varying stabilities. Common chiral cavity compounds encompass cyclodextrins, calixarenes, and crown ethers, which can accommodate guest molecules, facilitating chiral identification through the interaction force between the host and guest molecules. Numerous electrochemical sensors based on the selective matching of host and guest molecules have been developed, enabling the efficient differentiation of enantiomers. Yi et al. [58] constructed a dual-signal electrochemical chiral sensor for the highly sensitive detection of phenylalanine enantiomers (L/D-Phe) based on the mechanism of competitive host–guest interaction (Figure 2B). β-CD was electrooxidized on the surface of an electrode modified with carbon nanotubes@reduced graphene oxide (CNTs@rGO), with a hydrophobic cavity that can selectively capture rhodamine B (RhB) probe molecules to form a stable host–guest complex. The L-Phe has a significantly greater binding affinity with β-CD than that of RhB, promoting the incorporation of L-Phe into the β-CD cavity to replace RhB, which therefore reduces the oxidation peak current of RhB and produces the oxidation peak current of L-Phe. In contrast, D-Phe cannot compete for binding because of a spatial configuration mismatch, achieving the specific recognition of D/L-Phe, with a low LOD of 0.08 µM for L-Phe. The experimental results show that the dual-signal strategy (|ΔI_RhB_| + |ΔI_L-Phe_|, where ΔI denotes the change in peak current value) considerably improved the detection performance and enabled the quantitative detection of L-Phe in the racemic mixture, thereby affirming the critical role of the synergistic effect arisen from host–guest structure matching and dynamic substitution in the chiral recognition.

Molecular imprinting technology (MIT), characterized by a “lock and key” model, is an effective technique for forming polymers with specific recognition sites. The essence of molecular imprinting lies in the creation of functionalized complexes through interactions of functional monomers and template molecules, followed by the polymerization of these complexes under appropriate initiation conditions using crosslinking agents. Subsequently, the template molecules are extracted using physical or chemical methods, resulting in the preparation of molecularly imprinted polymers (MIPs) with a three-dimensional network structure [89]. The resultant MIP preserves cavities that closely resemble the shape, structure, and size of the template molecules, enabling selective recognition of these template molecules. However, the collapse of the imprinted cavities occurs during the elution of the template molecules, which can be effectively minimized by integrating the MIP with a metal–organic framework (MOF). Li et al. [90] prepared a MOF-based MIP electrochemical chiral sensor (Cu/Zn-BTC-MIP) through electropolymerizing MIP on MOF-modified electrodes. As shown in Figure 2C, a Cu/Zn-1,3,5-benzenetricarboxylic acid (Cu/Zn-BTC) MOF was electrodeposited on a glassy carbon electrode (GCE), followed by the electropolymerization of functionalized complexes assembled by the monomer (*o*-aminophenol) and template molecule (levamisole), to form the MIP with an imprinted cavity that exhibits a high degree of matching with levamisole on the electrode surface. Therefore, levamisole can be specifically adsorbed into the imprinted cavity, hindering the redox reaction of the probe [Fe(CN)_6_]^3−/4−^ on the electrode surface, thereby reducing the current response. However, dexamisole was precluded from entering the imprinted cavity due to spatial structure incompatibility, thus achieving the specific chiral recognition of levamisole with a considerably low LOD of 1.65 × 10^−12^ M (levamisole).

The structures of biological macromolecules contain many specific chiral molecules and different types of functional groups, which can interact with enantiomers to generate diastereomeric complexes, thereby effectively recognizing them. For example, BSA possesses a complex and stable three-dimensional conformation, with hydrophobic cavities and numerous functional groups on its surface, making it a commonly used chiral selector. Lai et al. [70] constructed an efficient chiral sensing interface (APS-DPANI-BSA/GCE) based on ammonium persulfate-doped polyaniline (APS-DPANI) and BSA for the detection of D/L-Trp. Figure 2D demonstrates that APS-DPANI was combined with BSA through a straightforward adsorption approach to form a composite material, which was modified on a GCE. The outcomes of DPV tests indicate that the sensing interface can preferentially bind to L-Trp for the effective recognition of Trp enantiomers, which is attributed to the different number of hydrogen bonds formed between the sensing interface and L/D-Trp. The calculated stability constants and Gibbs free energy values further confirm that the sensor binds a greater amount of L-Trp compared to D-Trp, leading to a lower peak current response for L-Trp than for D-Trp (I_D-Trp_/I_L-Trp_ = 1.95). Acceptable LODs of 0.071 mM and 0.0478 mM for L-Trp and D-Trp can be realized. Combining the excellent conductivity of APS-DPANI and the chiral recognition property of BSA, the sensor exhibited an excellent recognition efficiency (I_D-Trp_/I_L-Trp_) of 1.95 and was also successfully applied to the chiral recognition of Trp enantiomers in racemic mixtures.

### 2.2. Methods of Electrochemical Chiral Recognition

Electrochemical chiral recognition is based on different stereoselective interactions between chiral selectors modified on the electrode surface and the enantiomers, causing specific changes in redox currents, potentials, or interfacial impedances. The changes are converted to a readable electrical signal utilizing a conversion element and a signal amplifier. Depending on the electrical signal, commonly employed methods for electrochemical chiral recognition are differential pulse voltammetry (DPV), cyclic voltammetry (CV), electrochemical impedance spectroscopy (EIS), and square wave voltammetry (SWV).

**Figure 3:**
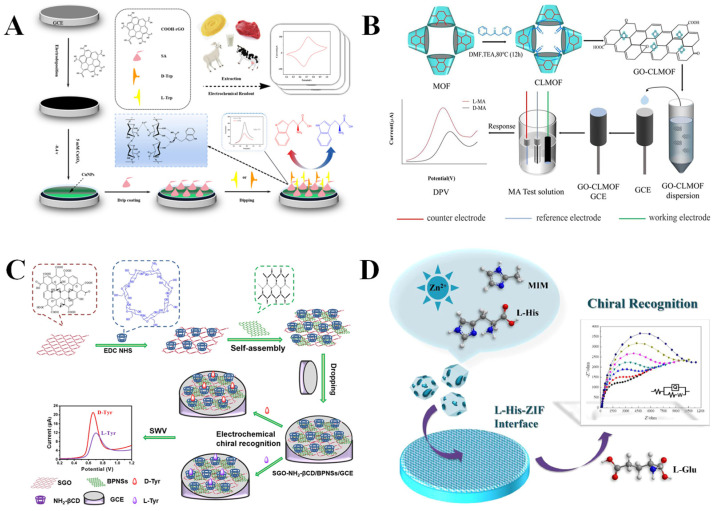
(**A**) Chiral recognition of Trp enantiomers using the CV method [59]. (**B**) Chiral recognition of MA enantiomers using the DPV method [60]. (**C**) Chiral recognition of Tyr enantiomers using the SWV method [61]. (**D**) Chiral recognition of Glu enantiomers using the EIS method [46].

CV is a method for inducing redox reactions in electroactive substances through applying a triangular wave potential to the electrode surface and simultaneously monitoring the current variations. The CV-based electrochemical chiral sensor enables the quantitative detection of enantiomers in real samples. For example, Wang et al. [59] constructed an electrochemical sensor based on rGO, copper nanoparticles (CuNPs), and sodium alginate (SA), achieving the quantitative detection of L/D-Trp in food samples by the CV method (Figure 3A). The rGO and CuNPs were electrodeposited on the electrode surface, followed by the dropwise addition of SA. The rGO and CuNPs can enhance the electron transfer efficiency on the electrode surface, while the inherent chiral structure of SA may bind specifically to the Trp enantiomers. In addition, the coordination between CuNPs and SA can improve the stability and conductivity of the electrochemical system. Ultimately, based on the relationship between peak current and concentration, the sensor achieved the quantitative detection of Trp enantiomers in beef, millet, goat milk, and cow milk. The sensor has low LODs for L-Trp (0.205 µM) and D-Trp (0.319 µM), respectively, with linear ranges of 5–500 µM. In reality, CV is mainly used to achieve chiral recognition through monitoring the alterations in redox peak currents [91] and potentials [92].

DPV uses the sum of a linear or step potential and an asymmetric pulse of fixed amplitude as the excitation signal. By performing a differential operation on the current at the end and the starting point of the pulse, the background noise can be significantly suppressed, and the detection sensitivity can be increased. For example, Sun’s group [60] developed a sensor based on a chiral cross-linked MOF (CLMOF) to achieve the chiral recognition of mandelic acid enantiomers (L/D-MA) using the DPV method (Figure 3B). The MOF was synthesized through the coordination of β-CD (organic ligand) with K^+^, which were then linked together to obtain CLMOF. Furthermore, graphene oxide (GO) and CLMOF were co-modified on the electrode to fabricate GO-CLMOF/GCE electrochemical sensors with enhanced conductivity. The MA enantiomers generated diastereoisomers with the CLMOF via hydrophobic interactions, host–guest recognition, and hydrogen bonding. The D-MA exhibited a more favorable interaction with CLMOF, leading to distinct electrochemical signals for efficient recognition of MA enantiomers with a peak current ratio (I_L-MA_/I_D-MA_) of 1.8 and a peak potential difference ΔE_p_ (E_D_–E_L_) of 96 mV. The acceptable LODs of 0.09 mM for L-MA and 0.15 mM for D-MA were obtained by the GO-CLMOF/GCE. MA is an α-hydroxycarboxylic acid that plays an important role in the pharmaceutical industry and the development of natural products. R-MA has been applied for the synthesis of antibacterial antibiotics, and S-MA is a raw material for manufacturing an anticholinergic drug (S-oxybutynin) to treat diseases related to urinary flora imbalance [93]. Therefore, THE development of efficient and sensitive methods for detecting MA enantiomers is significantly important.

SWV superimposes a series of forward and reverse symmetrical square wave pulse signals on the staircase potential ramp. During each square wave cycle, the current is measured twice, and the difference between the two measurements gives the differential current curve. Compared with DPV, SWV offers a shorter scan time and higher sensitivity [94]. For example, Zou et al. [61] covalently linked aminated β-CD (NH_2_-βCD) with a single-layer GO (SGO) and then successfully self-assembled it with black phosphorus nanosheets (BPNs) to construct a novel chiral sensing interface (SGO-NH_2_-βCD/BPNs) for the chiral recognition of tyrosine enantiomers (L/D-Tyr) using the SWV method (Figure 3C). In comparison with L-Tyr, the chiral sensing interface showed a reduced spatial site resistance with D-Tyr, indicating a more favorable stereoselective configuration of D-Tyr. Therefore, the sensor demonstrated a greater intermolecular interaction with D-Tyr, thus exhibiting a higher affinity for it. As a result, the reaction of the chiral sensing interface with D-Tyr showed a higher oxidation peak current with a lower oxidation peak potential. The results show that the sensing interface can sensitively identify Tyr enantiomers with low LODs (1.74 μM for L-Tyr, 1.02 μM for D-Tyr).

EIS measures the ratio of current to potential (impedance) or the impedance phase angle versus frequency through applying a sinusoidal alternating current potential of varying frequency with a small amplitude to a system. EIS is frequently employed to analyze the kinetics of electrode processes, bilayers, and diffusion. The interaction of the chiral selectors on the electrode surface with the enantiomer typically induces a variation in the impedance at the solid–liquid interface, which can be sensitively detected and amplified by EIS to realize the analysis of the target substance. For example, Wei et al. [46] reported a novel L-Histidine-zeolite imidazole framework (L-His-ZIF)-modified GCE through a simple drop-coating method, which enabled efficient and sensitive detection of glutamate enantiomers (L/D-Glu) using the EIS method (Figure 3D). The introduction of L-His makes the composite material possess a unique nanopolyhedral structure with a high specific surface area and cavities, forming a chiral environment and abundant chiral recognition sites. The specific interaction between L-His-ZIF and L-Glu led to an elevated impedance at the interface due to the poor conductivity of L-Glu, whereas D-Glu did not cause a notable change in impedance. Accordingly, enantioselective recognition and the quantitative detection of L-Glu were accomplished with a considerably low LOD (0.06 nM). It is worth noting that L-His-ZIF can be utilized for chiral recognition based on impedance changes, eliminating the necessity for additional conductive materials, hence streamlining the process. L-Glu, as an excitatory neurotransmitter of the vertebrate nervous system, is helpful for nerve activation. D-Glu is found in the capsule and cell wall of bacteria, and its effects on the human body are not clear [95]. Thus, the chiral discrimination of Glu enantiomers is conducive to the study of its different effects.

## 3. Biomaterials as Chiral Selectors for Electrochemical Chiral Recognition

### 3.1. Amino Acids and Their Derivatives

Amino acids [96] are a group of amphipathic organic small molecules characterized by the presence of both basic amino groups and acidic carboxyl groups, capable of forming peptides and proteins via peptide bonds. Amino acids can be mainly divided into α-, β-, and γ-amino acids according to the different positions of the amino group on the carbon chain, and α-amino acids are the constituents of natural proteins. Except for glycine (Gly), the α-carbon atoms of the remaining α-amino acids exhibit optical activity [97]. Therefore, amino acids and their derivatives can provide abundant chiral interaction sites to bind with enantiomers through non-covalent interactions, frequently serving as chiral selectors. They can be combined with carbon-based materials, a MOF, and metal nanoparticles (MNPs) to construct electrochemical chiral sensors for enantiomer recognition (Table 1). The three-point interaction model is often employed to elucidate the recognition mechanism of such sensors [98].

**Figure 4:**
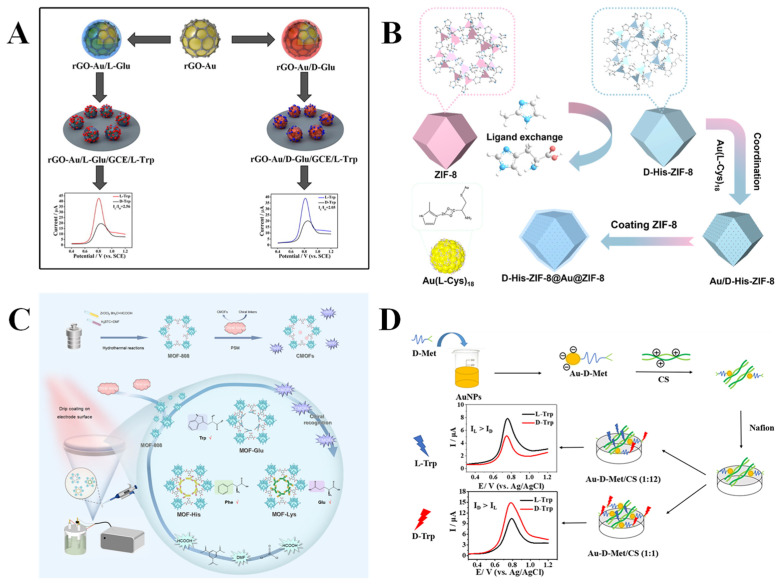
(**A**) Schematic diagrams of the sensor based on rGO-Au/Glu for identifying Trp enantiomers [35]; (**B**) the sensor based on D-His-ZIF-8@Au@ZIF-8 for identifying Phe enantiomers [38]; (**C**) the sensor based on CMOFs for identifying Trp, Phe, and Glu enantiomers [41]; and (**D**) the sensor based on Au-D-Met/CS for identifying Trp enantiomers [45].

Recently, researchers have been working on constructing electrochemical chiral sensors through combining amino acids and their derivatives with carbon-based materials. Carbon-based materials have excellent electrical properties, large surface areas, and commendable stabilities, which can significantly improve the recognition ability of electrochemical chiral sensors. For example, graphene, a two-dimensional carbon-based material with a honeycomb lattice, exhibits exceptional electrical conductivity and mechanical properties. It can be modified to obtain GO and rGO, which demonstrate enhanced performances and are extensively used in the construction of electrochemical sensors. Tartrate (Tart) has wide applications in food and pharmaceutical fields, such as beverage additives and acidifying agents [99]. The chiral recognition of Tart enantiomers is expected as the L-Tart and D-Tart have different physiological activities. Huang et al. [34] synthesized a chiral GO (L/D-GO) for the recognition of sodium tartrate (L/D-Tart) through covalently attaching cysteine (L/D-Cys) to the GO surface using a one-step hydrothermal process. The findings show that the chiral GO exhibits an excellent enantioselective recognition ability for Tart molecules with the same chirality. Interestingly, the synthesized chiral GO exhibited remarkable stability across a broad range of temperatures (20–200 °C) and pH levels (0–14), attributed to the robust covalent linkage between Cys and GO. Mo’s group [35] noncovalently modified gold nanoparticles (AuNPs) on the GO surface to obtain rGO-Au, which was functionalized with L/D-Glu to produce rGO-Au/Glu composites for the efficient chiral recognition of Trp enantiomers (Figure 4A). The rGO-Au/L-Glu showed a high peak current ratio (I_L-Trp_/I_D-Trp_) of 2.56 and acceptable LODs for L-Trp and D-Trp detection (0.28 mM and 0.86 mM, respectively), illustrating its excellent chiral recognition ability. L-Trp is an important precursor of protein and serotonin synthesis, which participates in the regulation of emotion and sleep. The lack of L-Trp will lead to mental diseases, such as anxiety and depression. D-Trp is a non-protein amino acid, which can be used in drug synthesis but cannot be metabolized by the human body [100]. Therefore, it is of great importance for the identification of Trp enantiomers.

The structure and properties of graphene can be further modulated through doping with heteroatoms (B, N, S, and others) for better electrochemical activity and a larger specific surface area [101,102]. For example, nitrogen-doped graphene quantum dots (NGQDs) were synthesized utilizing concentrated nitric acid and three-dimensional network graphene. Then, novel chiral sensing interfaces (L-NGQDs and D-NGQDs) were constructed through covalently attaching L/D-Cys to the surface of NGQDs using an amidation reaction, which were used for the electrochemical chiral recognition of L/D-Tart enantiomers [36]. The synergistic effect of the superior conductivity and extensive specific surface area of NGQDs, as well as the chiral structure of cysteine, makes the L-NGQD-modified electrode exhibit a significantly higher peak current signal for D-Tart, while the D-NGQD-modified electrode shows a more pronounced electrochemical response signal for L-Tart. The directly modified amino acids on the surface of carbon-based materials can produce chiral materials, whose chiral recognition sites are dependent on the amino acid itself. It is worth noting that related reports have demonstrated that the self-assembly of achiral substances and chiral templates can endow them with chirality, resulting in chiral composites with significantly enhanced chiral recognition effect. Niu et al. [37] prepared chiral templates (L/D-CQDs) using L/D-Trp as chiral source and attached them to carbon quantum dots (CQDs) through an amidation reaction. Then, the chiral composites (L/D-CQDs/TCPP) were obtained through the self-assembly of TCPP, promoted by hydrogen bonding and π–π interactions between the chiral templates and the achiral porphyrin (TCPP). The recognition of phenylalanine enantiomers (L/D-Phe) at L/D-CQDs/TCPP-modified GCE was performed using the DPV method, achieving high current ratios of 2.7 (I_D-Phe_/I_L-Phe_, using D-CQDs/TCPP/GCE) and 2.3 (I_L-Phe_/I_D-Phe_, using L-CQDs/TCPP/GCE), which were significantly higher than that of chiral CQDs alone. In brief, the intrinsic chirality of Trp can be transferred to the TCPP self-assembled structure by CQDs, forming a three-dimensional chiral interface with increased recognition sites and steric hindrance, thus effectively enhancing the enantioselectivity.

The applications of MOF materials in the study of using amino acids and their derivatives as chiral selectors have garnered increasing attention due to the designability, large specific surface area, and diverse structures of MOFs. The incorporation of amino acids and their derivatives into MOFs or their use as functional motifs to modify MOFs imparts a distinctive chiral microenvironment, which provides the possibility of efficient and selective electrochemical chiral recognition. For example, through directly incorporating amino acids as ligands to the MOF, Wang’s group [32] constructed an electrochemical sensor (KB/D-His-ZIF-8) for the chiral recognition of Trp enantiomers. The chiral recognition unit L-His in situ replaced 2-methylimidazole for coordination with Zn^2+^ to endow chirality to ZIF-8, and then Ketjen Black (KB) was incorporated to enhance the conductivity of the composite material. The efficient recognition of Trp enantiomers (I_L-Trp_/I_D-Trp_ = 5.78) was realized using GCE modified with KB/D-His-ZIF-8 via the drop-casting method. This sensor exhibited a high sensitivity of low LODs of 0.51 µM and 0.23 µM for L-Trp and D-Trp, respectively. The experimental results show that KB/D-His-ZIF-8 has the best affinity for L-Trp, resulting in more L-Trp being adsorbed on the electrode surface, but the electrochemical signal of Fe(CN)_6_^4−/3−^ is weakened due to the poor conductivity of L-Trp. L-Phe is involved in the synthesis of human neurotransmitters and is also a precursor for the synthesis of thyroxine and melanin. Its deficiency will cause phenylketonuria that harms the brain and intellectual development [103]. Further integrating MNPs with a MOF, Niu et al. [38] developed a sandwich electrochemical sensor (D-His-ZIF-8@Au@ZIF-8), which can be used for the chiral recognition of Phe enantiomers (I_L-Phe_/I_D-Phe_ = 1.97). As shown in Figure 4B, the sandwich structure can effectively overcome the problem of MNPs tending to agglomerate and detach from the outer layer of MOFs, leading to the effective enhancement of chiral recognition, as well as acceptable LODs of 0.195 mM and 0.588 mM for L-Phe and D-Phe, respectively. In another MOF-based sensor [39] D-His-ZIF-8@CoFe-PDA-modified GCE, the Co^2+^ and Fe^2+^-coordinated polydopamine (CoFe-PDA) significantly improved the electrochemical behavior of the sensing interface and augmented the chiral recognition sites, resulting in the effective recognition of Trp enantiomers and acceptable LODs of 0.066 mM and 0.15 mM for L-Trp and D-Trp, respectively. Although chiral MOFs (CMOFs) constructed directly from amino acids as ligands are comparatively straightforward to manipulate, the reaction process poses challenges in achieving perfect control. Therefore, providing a chiral environment through post-modifying the amino acid or its derivatives on the synthesized MOF is another effective strategy. Wang’s group [40] synthesized achiral PCN-224 (consisting of zirconium clusters with porphyrin ligands) via the solvothermal method, and grafted L/D-His to the surface of a MOF through covalent coupling to form the chiral material L/D-PCN-224, which was applicable for the chiral identification of Trp enantiomers. The spatial site-blocking effect of histidine created different chiral microenvironments around the original catalytic site of the MOF. The L-PCN-224 exhibited a higher current response to D-Trp (I_D-Trp_/I_L-Trp_ = 2.72), while D-PCN-224 produced a more enormous current response to L-Trp (I_L-Trp_/I_D-Trp_ = 2.68). Density-functional theory calculations further suggest that L-PCN-224 shows a greater affinity for D-Trp, while D-PCN-224 binds more readily to L-Trp. Meanwhile, the research group [41] constructed electrochemical sensors based on CMOFs (MOF-Glu, MOF-His, MOF-Lys) with varying chiral channel sizes through a post-synthesis modification strategy for the differentiation of L/D-Trp, L/D-Phe, and L/D-Glu, respectively (Figure 4C). The peak current of L-Trp was stronger than that of D-Trp, with a ratio of 2.69. Amino acids can also directly interact with MOF precursors to induce the formation or transformation of the MOF, hence altering their crystal structure or morphology and conferring chiral recognition functions to the materials. Based on the arrangement of ZIF-67 nanocrystals on the surface of L-Glu terminated bolaamphiphile (h-HDGA) to form a helical space structure, Zhao et al. [42] reported a chiral sensor (h-HDGA@ZIF-67/GCE) to realize the chiral analysis of penicillamine enantiomers (L/D-Pen). The oxidation peak potential difference (ΔE_p_) of h-HDGA@ZIF-67/GCE for Pen enantiomers reached 272 mV with low LODs of 0.022 μM (L-Pen) and 0.015 μM (D-Pen), enabling the quantitative detection of L-Pen in the racemic mixture. In addition, contact angle experiments reveal that the h-HDGA@ZIF-67 sensing interface has a greater affinity for L-Pen, resulting in easier access to the sensing interface and catalytic oxidation of L-Pen. In reality, the Pen enantiomers have different physiological effects on humans. D-Pen can treat heavy metal poisoning and rheumatoid arthritis, while the toxicity of L-Pen can cause symptoms such as neuritis and osteomyelitis [104].

In addition, amino acids and their derivatives are often combined with MNPs to construct electrochemical chiral sensors. AuNPs are extensively utilized owing to their ease of preparation, adjustable particle size, excellent biocompatibility, and good electrical conductivity. Mo et al. [43] developed an MIP based on AuNPs to achieve efficient detection of Trp enantiomers. Chiral molecularly imprinted AuNPs were obtained through co-electrodepositing chloroauric acid and template molecules (L/D-Trp) onto the electrode surface. The chiral microenvironment retained on the surface of AuNPs effectively enhanced the recognition of L-Trp and D-Trp with enantioselectivities of 2.788 (I_L-Trp_/I_D-Trp_) and 3.186 (I_D-Trp_/I_L-Trp_), respectively, as well as realized low LODs (0.012 µM and 0.009 µM for L-Trp and D-Trp, respectively). It is well known that cysteine contains sulfhydryl groups, which can bind to AuNPs through the Au–S bond. Therefore, Deng et al. [44] constructed a novel chiral sensor for distinguishing Trp enantiomers through modifying the surface of GCE with AuNPs and L-Cys complex (Au@p-L-Cys) via a simple electropolymerization method. With the assistance of Cu^2+^, L-Cys can form a complex with D-Trp, impeding electron transfer and leading to the reduction in current signal, whereas D-Trp is less prone to forming such a complex. The considerably low LOD for D-Trp is 75 nM, with a linear range of 6.1 × 10^−7^–1 × 10^−2^ M using the DPV method. Density-functional theory calculations indicate that the sensing interface has a high affinity to D-Trp, which was further verified by UV-visible spectral analysis. Although some studies have achieved chiral recognition through introducing a single chiral amino acid, the orientation of chirality is usually fixed, constraining the flexible regulation of the sensing interface. Notably, Chen et al. [45] reported a composite material (Au-D-Met/CS) based on AuNPs, D-methionine (D-Met), and chitosan (CS) for the recognition of Trp enantiomers, achieving considerably low LODs of 42.16 pM for L-Trp and 7.75 pM for D-Trp, respectively (Figure 4D). Circular dichroism (CD) spectroscopy showed that Au-D-Met/CS (1:1) displayed a positive signal predominantly attributed to D-Met, whereas Au-D-Met/CS (1:12) exhibited a negative signal primarily derived from CS. Therefore, through adjusting the ratio of D-Met to CS, the chiral orientation of Au-D-Met/CS can be altered. The DPV test results indicate that L-Trp and D-Trp displayed opposite peak current signals on electrodes modified with Au-D-Met/CS (1:12, I_L-Trp_ > I_D-Trp_) and Au-D-Met/CS (1:1, I_D-Trp_ > I_L-Trp_), respectively. Recently, researchers have been more devoted to exploring the mechanism of electrochemical chiral recognition. Bodoki’s group [33] deposited L/D-Cys-modified AuNPs on GCE to construct a sensor (L/D-Cys-AuNPs) and investigated the mechanism of the interaction between the sensor and propranolol (PRNL) from the thermodynamic and kinetic perspectives using CV, DPV, EIS, and isothermal titration calorimetry (ITC). The ITC results indicate that L-Cys-AuNPs displayed enantioselectivity for PRNL and exhibited a more robust interaction with S(-)-PRNL. However, D-Cys-AuNPs cannot discriminate between R(+)-PRNL and S(-)-PRNL. In addition, the heterogeneous electron transfer rate (*k*_0_) of L-Cys-AuNPs for S(-)-PRNL (5.2394 cm/s) exceeds that for R(+)-PRNL (4.1048 cm/s), suggesting preferential adsorption and enhanced charge transfer, while the difference between the *k*_0_ of D-Cys-AuNPs for S(-)-PRNL (5.5902 cm/s) and R(+)-PRNL (5.5608 cm/s) is negligible. It should be mentioned that PRNL is an adrenergic receptor blocker used to treat arrhythmia, angina pectoris, and hypertension. The β-receptor blocking effect of S(-)-PRNL is approximately 100 times stronger than that of R(+)-PRNL [105].

### 3.2. Polysaccharides and Their Derivatives

Polysaccharides are biomolecules consisting of multiple monosaccharide molecules (ten or more) linked by glycosidic bonds, which are prevalent in plants, animals, and microorganisms [106]. They possess abundant functional groups, including hydroxyl and carboxyl groups, which can form complexes with chiral molecules through various non-covalent interactions, such as hydrogen bonding, van der Waals forces, and hydrophobic interactions [20]. Furthermore, the complex helical structure of polysaccharides can provide a chiral microenvironment for chiral recognition, thus realizing the separation of enantiomers. Polysaccharides used as chiral selectors generally include chitosan (CS) [107], cellulose [51], sodium alginate (SA) [108], and starch [109], which have widespread availability and biocompatibility.

**Figure 5:**
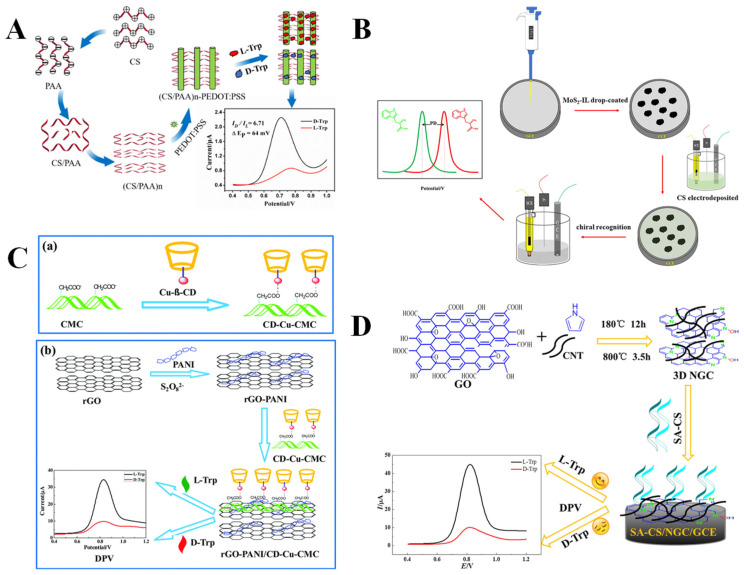
(**A**) Schematic diagrams of the sensor based on (CS/PAA)n@PEDOT:PSS for identifying Trp enantiomers [48]; (**B**) the sensor based on MoS_2_-IL/CS for identifying Trp enantiomers [49]; (**C**) the sensor based on rGO-PANI/CD-Cu-CMC for identifying Trp enantiomers [53]: (a) the synthesis of chiral selector CD-Cu-CMC, (b) the synthesis of chiral composite rGO-PANI/CD-Cu-CMC; and (**D**) the sensor based on SA-CS-NGC for identifying Trp enantiomers [56].

CS is a naturally occurring alkaline polysaccharide derived from the deacetylation of chitin, which has the advantages of a good film-forming property, strong adhesion, and an excellent mechanical property [110]. CS has chiral characteristics owing to its abundant chiral carbon atoms and unique helical conformation (comprising six sugar units per helix) in the molecular structure, leading to its extensive research in electrochemical chiral recognition [111]. Different methods have been employed to complex CS with other materials, including electrodeposition, self-assembly, and covalent binding, thereby enhancing the sensors’ chiral recognition and electrochemical performance. For example, Gong et al. [47] constructed an electrochemical sensor (Cu_2_-β-CD/NH_2_-CS-MWCNTs) with renewability through a coordination-driven self-assembly of aminated CS-MWCNTs (NH_2_-CS-MWCNTs) with Cu^2+^-modified β-CD (Cu_2_-CD) on the electrode surface. The smaller spatial site resistance of L-Trp promoted its penetration through the interface for oxidation, resulting in the chiral recognition of Trp enantiomers via the DPV method, with a peak current ratio (I_L-Trp_/I_D-Trp_) of 1.41 and a peak potential difference (ΔE_p_) of 35 mV as well as acceptable LODs of 13.4 µM and 18.5 µM for L-Trp and D-Trp, respectively. Interestingly, the electrochemical chiral recognition ability of the sensor can be restored through electrochemical cleaning and re-self-assembly. In addition, the layer-by-layer self-assembly technique is a method for constructing multilayer sensing interfaces with significant tunability. Yang et al. [48] fabricated multilayer chiral membranes ((CS/PAA)n) through the sequential deposition of CS and polyacrylic acid (PAA) on GCE, subsequently integrating the chiral membranes with conductive poly (3,4-ethylenedioxythiophene): poly (styrenesulfonate) (PEDOT:PSS) to develop chiral sensors (Figure 5A). The sensor ((CS/PAA)n@PEDOT:PSS/GCE), constructed based on the layer-by-layer self-assembly of oppositely charged substances, can distinguish Trp enantiomers efficiently and obtain low LODs of 0.33 µM and 0.67 µM for L-Trp and D-Trp, respectively. Despite the self-assembly method being easy to operate and having favorable uniformity of film formation, the assembly process is time-consuming. In reality, the fabrication of chiral interfaces through the electrodeposition of polysaccharides is an alternatively effective approach. Hou et al. [49] developed an electrochemical chiral sensor based on a composite formed by the electrodeposition of CS on GCE modified with molybdenum disulfide-ionic liquid (MoS_2_-IL) for highly selective recognition of Trp enantiomers (Figure 5B). Through optimizing the electrodeposition parameters, rapid and uniform formation of CS film was accomplished, and the DPV assay showed that the peak potential difference (ΔE_p_) between L-Trp and D-Trp reached 53.3 mV, a more than 13-fold enhancement over the electrode with unmodified CS (ΔE_p_ = 4 mV). Unlike the self-assembled and electrodeposited non-covalent linkage approaches, Gou et al. [50] prepared a composite (RGO/CS) through covalently anchoring CS on the surface of RGO via an amidation reaction for the chiral recognition of Tyr enantiomers. The results show that RGO/CS is preferentially bound with L-Tyr of small spatial site resistance, resulting in the formation of diastereoisomers, and weakening the current signal. Conversely, D-Tyr exhibits significant spatial site resistance, making it difficult to establish three-point interactions with RGO/CS. The recognition of Tyr enantiomers is considered necessary. L-Tyr is an important precursor for synthesizing biologically active substances such as melanin, adrenaline, dopamine, etc. Insufficient levels of L-Tyr will lead to depression, anxiety, and Parkinson’s disease. In contrast, D-Tyr is used as a raw material for the manufacture of various drugs [112].

Cellulose is a natural polymer consisting of D-glucose (D-Glc) units linked by β-1,4-glycosidic bonds and is widespread in plant cell walls [113]. Each glucose unit contains multiple chiral carbon atoms, causing cellulose to exhibit a high degree of enantioselectivity as a whole [114]. Therefore, cellulose and its derivatives show promising potential for their applications in chiral recognition sensing. Sodium carboxymethyl cellulose (CMC), which has reactive carboxyl groups, is often covalently coupled with other substances. Han et al. [51] prepared a composite material through covalently grafting CMC onto the surface of MOF (MIL-88(Fe)) via an amidation reaction, which was used to recognize Trp enantiomers using the DPV method. The results indicate that L-Trp can penetrate the membrane structure of CMC due to its steric configuration matching the glucose unit of CMC, whereas D-Trp shows a significantly diminished binding ability caused by a large steric hindrance, resulting in a chiral recognition efficiency of 2.04 (I_L-Trp_/I_D-Trp_). Researchers have also combined CMC with conductive materials through electrostatic interactions. Du et al. [52] developed an electrochemical chiral sensor (rGO-PhenCu-CMC/GCE) through combining a substrate material rGO-Phenanthroline copper (rGO-PhenCu) with CMC utilizing electrostatic interactions. Based on the different spatial site resistance effects of L/D-Trp versus rGO-PhenCu-CMC/GCE, the sensor exhibited a greater affinity for L-Trp, resulting in different peak currents (I_L-Trp_/I_D-Trp_ = 2.41). Interestingly, the synergistic self-assembly among polysaccharides has beneficial effects in addressing their agglomeration problem. For example, copper-coordinated β-CD (Cu-β-CD) was combined with CMC through an electrostatic attraction to create a polysaccharide composite (CD-Cu-CMC), which was subsequently incorporated into the substrate material rGO-polyaniline (rGO-PANI) to establish an electrochemical chiral sensor (rGO-PANI/CD-Cu-CMC/GCE) for the chiral recognition of Trp enantiomers [53] (Figure 5C). Due to the effective inhibition of polysaccharide aggregation by the bis-polysaccharide complex (CD-Cu-CMC), the chiral recognition site of the sensor can be fully exposed, resulting in a DPV response current ratio (I_L-Trp_/I_D-Trp_) of 3.58.

SA is a naturally occurring anionic polysaccharide sourced from brown algae, which is composed of β-D-galacturonic acid (M unit) and α-L-guluronic acid (G unit) alternately linked by 1,4-glycosidic bonds [115]. The ratio and distribution of the M and G units in the molecular chain directly affect its physicochemical properties. The chirality of SA originates from the inherent chiral configuration of M and G units, which can be applied to construct electrochemical chiral sensors. Niu et al. [54] constructed an electrochemical sensor (CNT/PANI/SA/GCE) based on electrostatic-driven self-assembly through modifying a multi-walled carbon nanotube/polyaniline/SA (CNT/PANI/SA) composite on the surface of GCE. This strategy successfully combines the chiral recognition ability of SA with the conductivity of CNT/PANI, which was applied in the effective chiral recognition of Trp enantiomers. DPV test results show that the sensor has a higher affinity for L-Trp, and thus there is a significant difference between the peak currents of L-Trp and D-Trp (I_D-Trp_/I_L-Trp_ = 2.1). Given that CS acquires a positive charge when dissolved in glacial acetic acid, while SA becomes negatively charged upon dissolution in water, Pei et al. [55] combined CS and SA to form a composite material (CS-SA) using the electrostatic self-assembly method. The composite was then modified on GCE to obtain a chiral sensor (CS-SA/GCE) for the identification of Tyr enantiomers. When the mass ratio of CS and SA was 1:1, CS-SA exhibited a homogeneous porous structure with the best chiral recognition efficiency, and the current ratio (I_L-Tyr_/I_D-Tyr_) obtained from the SWV test was 1.63, with considerably low LODs of 29 nM (L-Tyr) and 107 nM (D-Tyr). In addition, the covalently bound two polysaccharides exhibit a sparse three-dimensional network structure, which can effectively reduce the agglomeration effect between the polysaccharides and improve the chiral recognition efficiency. Niu et al. [56] prepared the SA-CS through an amidation reaction between the amino groups of CS and carboxyl groups of SA, followed by mixing SA-CS with 3D nitrogen-doped rGO-CNT (3D NGC) and drop-casting the resultant mixture onto a GCE (SA-CS-NGC/GCE) for the electrochemical chiral recognition of Trp enantiomers (Figure 5D). The substantial steric barrier between SA-CS-NGC/GCE and L-Trp during the formation of diastereomeric complexes facilitated the penetration of L-Trp across the SA-CS membrane, leading to elevated peak current values (I_L-Trp_/I_D-Trp_ = 4.52). UV-visible spectroscopy analysis further indicates a lower binding constant between SA-CS and L-Trp.

### 3.3. Proteins

Proteins are naturally occurring macromolecular compounds that constitute the basic substances in living organisms, essential for growth and maintenance of life. They are formed by the condensation of twenty different L-α-amino acids through peptide bonding, and the main chain exhibits a regular primary structure, which is further folded into specific secondary (α-helix, β-fold), tertiary, and quaternary spatial configurations through non-covalent interactions, including van der Waals’ forces, hydrophobic interactions, and hydrogen bonding [116]. The unique steric structure of the proteins and the chiral nature of the contained subunits (L-α-amino acids) can create chiral recognition sites for enantiomer-specific binding. The primary proteins that have been investigated as chiral selectors are: (i) albumin (BSA and human serum albumin (HSA)) [117]; (ii) glycoprotein (α1-acid glycoprotein) [118]; (iii) oxygen-carrying proteins (hemoglobin) [119]; and (iv) immunoglobulins (γ-globulin) [120].

**Figure 6:**
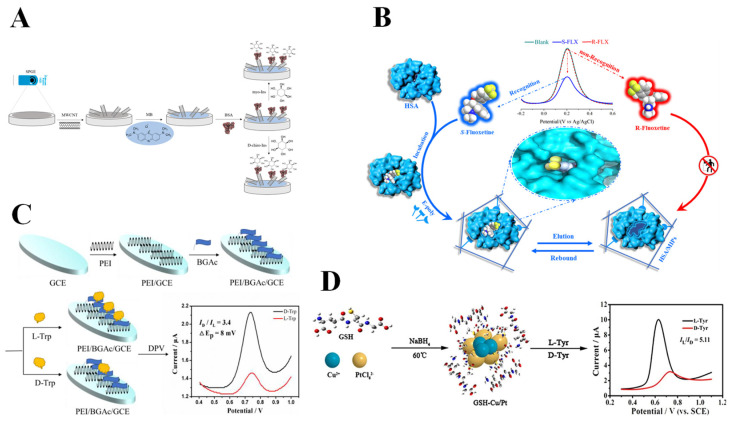
(**A**) Schematic diagrams of the sensor based on BSA/MB/MWCNT/GSPE for identifying myo-Ins and D-chiro-Ins [63], (**B**) the molecular imprinting sensor based on HSA for identifying S-FLX [66], (**C**) the sensor based on PEI/D-BGAc for identifying Trp enantiomers [67], and (**D**) the sensor based on GSH-Cu/Pt for identifying Tyr enantiomers [69].

Gamma-globulin (GLOB) is an important type of protein in human serum. Its core components are immunoglobulin (e.g., IgG, IgA, and IgM), which are produced by the immune system and are responsible for recognizing and neutralizing pathogens [121]. In the previous research, GLOB had been attempted as a recognition element for electrochemical chiral sensors [120,122,123]. Recent research has been increasingly focused on the application of albumin for electrochemical chiral recognition, particularly BSA. Owing to its structural stability, widespread availability, low cost, and simplicity of modification, BSA has emerged as the most representative protein selector in electrochemical chiral recognition. BSA is a globular protein composed of 583 amino acid residues, containing three helical domains (I, II, and III), and each domain can be divided into A and B subdomains [124]. The asymmetric arrangement and spatial folding of the domains form a three-dimensional heart-shaped structure with chiral microenvironments, enabling the separation of enantiomers. Inspired by the excellent biocompatibility of GQD and its capacity for functionalization with diverse biomolecules, Ye et al. [62] constructed an electrochemical chiral sensor using GQD-functionalized BSA for the chiral recognition of Trp enantiomers. The combination of GQD and BSA through an amidation reaction induced a change in the spatial structure of BSA, which exposed more chiral recognition sites on the surface of BSA, leading to a chiral identification of Trp enantiomers (I_L-Trp_/I_D-Trp_ = 3.67) using the DPV method. Density-functional theory calculations further elucidate that the chiral interface exhibits a higher affinity for L-Trp. Meanwhile, another carbon-based material, MWCNT, provides a distinct advantage for the noncovalent immobilization of BSA due to its large specific surface area, one-dimensional tubular structure, and high electrical conductivity. As shown in Figure 6A, Tortolini et al. [63] constructed an electrochemical sensing interface based on BSA and MWCNTs on the surface of graphite screen-printed electrodes, thereby realizing the effective recognition of Myo-inositol (myo-Ins) and D-chiro-inositol (D-chiro-Ins). The low LODs for myo-Ins and D-chrio-Ins are 0.5 µM and 1 µM, respectively. Molecular docking experiments show that the hydrophobic cavity of BSA binds to the inositol isomers through van der Waals forces and hydrogen bonding, with myo-Ins forming more hydrogen bonds due to the spatial orientation that better matches the hydrophilic sites of BSA. Myo-Ins and D-chiro-Ins have been studied for the treatment of polycystic ovary syndrome (PCOS), which can improve insulin resistance, enhance egg quality, and reduce the risk of gestational diabetes. The ratio of myo-ins and D-chiro-Ins in healthy women’s ovaries is about 100:1, while PCOS patients will deviate from this normal value [125]. Therefore, accurate identification of myo-ins and D-chiro-Ins is significant for biomedicine. In addition, BSA can be anchored to covalent organic framework materials (COFs) [64] or loaded on the surface of magnetic titanium dioxide nanomaterials [65] for chiral amino acid recognition. On the other hand, HSA is a single polypeptide chain of 585 amino acid residues that establishes a stable globular three-dimensional structure via 17 pairs of disulfide bonds. It exhibits structure similarity to BSA, containing three homologous structural domains, I, II, and III, each of which is further divided into two subdomains, A and B. The chiral substance binds to HSA mainly through Sudlow sites I and II, which are mainly located in the IA and IIA subdomains [124]. Zhang et al. [66] developed an MIP sensor (MIPs/HSA) for the detection of S-fluoxetine (S-FLX) using HSA as an auxiliary recognition unit (Figure 6B). The sensor stabilized the stereo conformation of the chiral molecule S-FLX through the natural chiral site of HSA, leading to an 18.5-fold increase in the selectivity coefficient and a considerably low LOD of 6.43 × 10^−17^ mol L^−1^ compared with the MIP sensor without HSA (MIPs/n). The results of UV-visible and fluorescence spectroscopy analysis and molecular simulation demonstrate that interaction modes between S-FLX and HSA are mainly van der Waals forces, hydrophobic interactions, and hydrogen bonding. As an antidepressant, FLX is used to treat depression, obsessive–compulsive disorder, and other mental diseases. Both S-FLX and R-FLX have inhibitory effects on serotonin reuptake. However, the metabolic clearance rate of R-FLX in the human body is four times more than that of S-FLX [126]. Thus, the development of rapid and sensitive enantiomer recognition methods can facilitate the efficient use of FLX.

Chiral peptides are a class of peptide compounds formed by the assembly of chiral amino acids via peptide bonds, which have the properties of structural stability, low cost, favorable biocompatibility, and excellent thermal stability [127]. Additionally, their facile modification and capacity for self-assembly with other materials have attracted considerable interest from researchers. Sun et al. [67] constructed a three-dimensional porous electrochemical chiral interface (PEI/D-BGAc/GCE) through the self-assembly of polyethyleneimine (PEI) and a chiral peptide (D-BGAc), achieving efficient recognition of Trp enantiomers (Figure 6C). L-Trp tends to form hydrogen bonds with chiral interfaces, but D-Trp is less susceptible to the interface due to steric hindrance, resulting in a high current ratio (I_D-Trp_/I_L-Trp_) of 3.4 and low LODs of L-Trp and D-Trp (0.67 µM and 0.33 µM, respectively) as detected by the DPV method. In addition, Glutathione (GSH) is a tripeptide composed of glutamate, cysteine, and glycine, commonly found in human cells. GSH sustains intracellular homeostasis via redox reactions of sulfhydryl groups (–SH), in addition to executing many physiological functions, such as heavy metal detoxification, anti-tumor activity, and immune regulation, which are important for maintaining normal cellular activity [128]. The molecular structure of GSH contains two chiral carbon atoms situated at the α-carbon sites of glutamate and cysteine, respectively, which play key roles in enantioselective binding. In reality, α-CD can be introduced to protect –SH of GSH from oxidation and enhance its chiral recognition ability. Leveraging the chiral properties of GSH and the hollow cavity structure of α-CD, Ye et al. [68] encapsulated GSH with α-CD, creating a host–guest complex (α-CD/GSH) to protect the –SH of GSH. The α-CD/GSH-modified GCE can be used for the chiral recognition of Trp enantiomers (I_L-Trp_/I_D-Trp_ = 3.88), with a recognition efficiency nearly twice as high as that of the GCE modified with GSH alone. Contact angle experiments, UV-visible analysis, and calculations of binding constants indicate that the constructed sensor exhibits a high affinity for L-Trp compared with D-Trp. It is worth noting that the –SH of GSH can form stable covalent bonds with specific MNPs. Kong’s group [69] simultaneously modified GCE with GSH, CuNPs, and PtNPs (GSH-Cu/Pt/GCE) for the identification of Tyr enantiomers using the DPV method (Figure 6D). The sensor exhibited a peak current ratio (I_L-Tyr_/I_D-Tyr_) of 5.11 and a peak potential difference (ΔE_p_) of 104 mV for Tyr enantiomers. The enhanced recognition efficiency of GSH-Cu/Pt/GCE toward Tyr enantiomers may be ascribed to the different quantities of hydrogen bonds formed between GSH and L-Tyr and D-Tyr, respectively.

### 3.4. Enzymes

**Figure 7:**
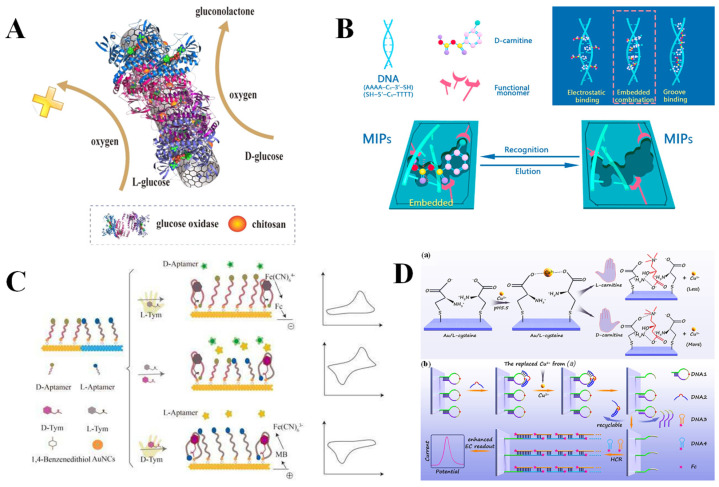
(**A**) Schematic diagrams of the sensor based on β-D-GOD@CS-CNTs for identifying Glc enantiomers [71], (**B**) the sensor based on MIPs/dsDNA for identifying D-carnitine [76], (**C**) the chiral aptamer sensor based on Apt@AuNCs for identifying Tym enantiomers [77], and (**D**) the sensor based on Cu^2+^-dependent DNAzyme for identifying carnitine enantiomers [79]: (a) the formation of Cu^2+^-L-Cys complexes, (b) the formation of DNAzyme cleavage reaction and hybrid chain reaction.

Enzymes are a category of biologically active macromolecules produced by living cells that exhibit stereospecific catalytic activity. The core function of enzymes is to reduce the activation energy of chemical reactions, thereby efficiently accelerating metabolic reactions within organisms. Their catalytic activity depends on specific spatial structures and can function under mild physiological conditions [129]. In addition to exhibiting weak interactions with chiral substances, enzymes catalyze chiral substrates through stereospecific chemical reactions, offering advantages such as high catalytic efficiency, pronounced specificity, and activity tunability. These properties render enzymes a promising option for constructing highly selective electrochemical chiral sensors. For instance, β-D-glucose oxidase (β-D-GOD) and D-amino acid oxidase (DAAO) utilize oxygen as an electron acceptor and specifically catalyze D-isomers to generate hydrogen peroxide and D-glucono-δ-lactone, hydrogen peroxide and imino acid, respectively [130,131]. Glc in human blood is an essential energy source to maintain the normal function of tissues and organs. Abnormal Glc levels can lead to diabetes, which seriously endangers human health [132]. Hence, accurate detection of Glc is meaningful for effectively preventing diseases. As illustrated in Figure 7A, Zhu et al. [71] reported an electrochemical chiral sensor based on β-D-GOD using CNT and CS as substrate materials (CNT-CS). β-D-GOD specifically catalyzed the oxidation of D-Glc to gluconolactone, consuming oxygen and producing a change in the reduction current signal. The sensor can effectively detect D-Glc (LOD = 0.085 mM) and achieve the quantitative analysis of D-Glc in complex samples. On the other hand, DAAO exhibits highly stereoselective catalytic activity toward D-amino acids, specifically promoting their conversion to the corresponding keto acids, hydrogen peroxide, and ammonia. Given the oxidative property of hydrogen peroxide, it can participate in redox reactions with various metals and metal oxides. Liu et al. [72] developed an electrochemical sensor for detecting alanine enantiomers (L/D-Ala) based on core–shell structured magnetic nanoparticles (Fe_3_O_4_@Au@Ag@Cu_x_O NPs) and DAAO. An increase in hydrogen peroxide concentration enhances the chemical oxidation of Cu, thereby diminishing the peak current of Cu’s electrooxidation. This sensor exhibits high sensitivity toward D-Ala, with a considerably low LOD of 52 pM. Notably, the redox center flavin adenine dinucleotide/flavin mononucleotide, reduced (FAD/FADH_2_) of DAAO can generate electrochemical signals through direct electron transfer processes on the electrode surface. In reality, L-Ala promotes the production of insulin, while D-Ala is generally not tolerated by living organisms [133]. Tian et al. [73] developed an electrochemical sensor utilizing CNTs and DAAO for the highly sensitive and selective detection of Ala enantiomers. As the concentration of D-Ala increased, the peak current gradually decreased, with a low LOD of 7.91 μM for D-Ala, and the quantitative detection of D-Ala in racemic mixtures was also achieved. The mechanism underlying the changes in peak current values was further discussed and validated through CV experiments. D-Ala consumes the oxidized state of DAAO (FAD) through a catalytic reaction and reduces the amount of reducible FAD on the electrode surface, resulting in a decrease in the reduction peak current. Unlike traditional voltametric detection methods, Muñoz et al. [74] directly immobilized L-amino acid oxidase (LAAO) on the surface of 3D-printed nanocomposite carbon electrodes (3D-nCE) to construct an electrochemical sensor. Utilizing the EIS method to identify alterations at the 3D-nCE interface caused by hydrogen peroxide generated from the reaction of L-Ala catalyzed by LAAO, the sensor achieved remarkable sensitivity for L-Ala detection, with a considerably low LOD of 10 fM.

### 3.5. Nucleic Acids

Nucleic acids are the key biomolecules responsible for storing and transmitting genetic information in living systems, including DNA and RNA [134]. DNA is a macromolecular polymer composed of bases, deoxyribose, and phosphate, typically exhibiting a right-handed helical structure and possessing a chiral center. Therefore, DNA can serve as a useful chiral selector in electrochemical chiral sensing [135]. Research [136] has shown that the recognition patterns between small molecules and DNA are: (i) groove binding: primarily occurring in major or minor grooves, driven by hydrogen bonding and van der Waals forces; (ii) intercalation binding: molecules insert between base pairs, stabilized by π-stacking and hydrophobic interactions; and (iii) electrostatic interactions: attraction between positively charged molecules and the polyionic phosphate backbone of DNA. The DNA-embedded MIP strategy can achieve highly selective recognition of chiral target molecules through embedding double-stranded DNA (dsDNA) as a conformational anchor unit within the MIP membrane [75]. Zhang et al. [76] constructed MIPs/dsDNA sensors through incorporating D-carnitine into dsDNA on a gold electrode surface through electropolymerization, enabling the quantitative analysis of D-carnitine using the DPV method (Figure 7B). The synergistic effect of the imprinted cavity and DNA chiral recognition resulted in a spatial conformation of the imprinted cavity that is highly matched with that of D-carnitine, realizing a considerably low LOD of 2.24 × 10^−16^ mol/L for D-carnitine. It is well known that aptamers are small-molecule oligonucleotides characterized by distinct three-dimensional structures with a high affinity and specificity for their targets [137]. An electrochemical biosensor based on aptamer-modified gold nanoclusters (AuNCs) utilized the dense insulating layer formed by AuNCs and the chiral aptamer (D-Apt/L-Apt) functionalized structure to achieve stereoselective signal amplification and the recognition of tyrosinamide enantiomers (L/D-Tym), with enantiomeric recognition efficiency as high as 4.0 [77] (Figure 7C). Tym is a derivative of Tyr, where the carboxyl group in Tyr is replaced by an amide group. As the simplest mimic of Tyr, Tym can regulate the topological structures of DNA within cells, which is of great significance in the research of enzyme catalytic activity [138]. It is worth noting that DNA can be integrated with natural polysaccharides to construct biocompatible chiral recognition interfaces. Sun et al. [78] employed layer-by-layer self-assembly technology to modify CS with DNA on GCE, constructing an electrochemical chiral sensor for the effective identification of Trp enantiomers. The sensor demonstrated a pronounced affinity for L-Trp, inhibiting its penetration to the electrode surface and resulting in a decrease in the oxidation peak current. DPV test results show an enantioselectivity recognition efficiency of 4.02 (I_D-Trp_/I_L-Trp_), significantly higher than that of the sensor modified with CS or DNA alone. The low LODs of the sensor for D-Trp and L-Trp are 1.33 μM and 1.67 μM, respectively. In addition, deoxyribonucleases (DNAzyme) are artificially synthesized DNA sequences with catalytic activity similar to that of proteases. They can catalyze specific biochemical reactions, including RNA cleavage, RNA ligation, and DNA cleavage [139]. Their catalytic activity depends on the specific three-dimensional spatial conformation formed by sequence folding and typically requires cofactors to stabilize their active sites [140]. DNAzyme offers several advantages, such as high specificity, good stability, low cost, and ease of synthesis and modification. Consequently, they have attracted widespread attention from researchers in the field of sensing [141]. Zhai et al. [79] constructed an electrochemical chiral sensor for identifying carnitine enantiomers using the DPV method based on Cu^2+^-dependent DNAzymes and Cu^2+^-amino acid complexes (Figure 7D). The heterochirality of D-carnitine with L-Cys displaced more Cu^2+^ than the homochirality of L-carnitine, promoting the DNAzyme to cut the substrate chain and release the trigger DNA, thereby initiating a hybrid chain reaction and achieving signal cascade amplification. The enantiomers of carnitine have opposite physiological effects. L-carnitine promotes fatty acids to enter mitochondria for oxidation and decomposition, thereby releasing energy, which is beneficial for treating chronic kidney diseases and cardiovascular diseases. However, D-carnitine is toxic and can cause fatigue and muscle atrophy [142]. Therefore, the distinction of carnitine enantiomers is necessary. In addition, L-His is one of the essential amino acids for the human body and is involved in various physiological processes. L-His can be converted into histamine, which participates in the immune system response. In comparison, D-His, as an intermediate of drug synthesis, does not possess biological activity [143]. Han et al. [80] constructed an on-off-on electrochemical sensor using iron oxide nanoparticles and reduced graphene oxide composite material (Fe_3_O_4_@rGO) as dual-signal probes and His-dependent DNAzyme as a catalytic system, realizing the recognition of His enantiomers. The dual-signal intensities caused by L-His are 2.61 and 2.68 times higher than that of D-His, showing an excellent chiral recognition ability, with considerably low LODs of 0.47 pM and 0.68 pM, respectively.

In summary, amino acids and their derivatives, polysaccharides and their derivatives, proteins, enzymes, and nucleic acids have been studied for electrochemical chiral sensing to recognize enantiomers, which show different characteristics. Table 2 shows the prominent advantages, disadvantages, detection object of different biological materials, as well as the lowest LOD and the highest chiral recognition efficiency they have achieved in the electrochemical chiral recognition process. (i) Amino acids and their derivatives have clear structures, appropriate molecular sizes, and are easy to form complexes with other materials (carbon-based materials, MOF, and MNPs) to obtain chiral composites with high recognition ability. However, a single amino acid provides few chiral sites, and the chiral recognition ability is weak. (ii) Polysaccharides are natural biological macromolecules with excellent biocompatibility, low toxicity, good hydrophilicity, and ease of modification. However, polysaccharides as chiral selectors also have some disadvantages, such as easy agglomeration and poor conductivity. At present, electrodeposition, covalent-grafting, and self-assembly have been used to solve these problems. (iii) All hierarchical structures of proteins are chiral, and their numerous chiral sites and hydrophobic cavities give them unique stereoselectivity, showing the advantages of rich functional groups and easy availability. However, proteins tend to be strongly adsorbed on the electrode surface, which may shield the redox active sites and reduce the electric signal. (iv) Enzymes exhibit high specificity and catalytic efficiency for substrates. The enantiomer recognition of electrochemical chiral sensors based on enzymes shows high specificity, sensitivity, and interference-resistance ability, which is suitable for complex systems. It should be noted that the temperature, pH, solvent, and other conditions have great influence on enzyme activity. (v) The right-handed double helix structure of DNA makes it a candidate for the chiral recognition of enantiomers. The natural DNA that has been applied in the field of electrochemical chiral recognition includes calf thymus double-stranded DNA [144], fish sperm double-stranded DNA [145], and human telomere DNA [27], but the chiral recognition ability of these natural DNAs is limited. In contrast, aptamers and DNAzymes show satisfactory biocompatibility, controllable self-assembly characteristics, and stable chemical properties. However, the uncertainty of DNA structure design limits its development in the field of sensing.

## 4. Discussion and Prospects

Chiral molecules are widely found in the natural environment and within living organisms, and enantiomers often exhibit different or even opposite physiological activities. Therefore, it is of crucial importance to accurately identify enantiomers in biological systems as well as to effectively separate and quantify them in ecosystems. Compared with traditional chromatographic and spectroscopic methods, electrochemical analysis is a highly sensitive, selective, and versatile technique for chiral recognition, offering several advantages, such as simplicity of operation and rapid response. Various electroanalytical techniques (CV, LSV, SWV, DPV, and EIS) have been used for the electrochemical chiral recognition of different enantiomers. CV is mainly employed to obtain quantitative information in the electrochemical process, including oxidation and reduction processes on the electrode surface, oxidation and reduction potentials of electroactive species, the reaction mechanism, and reversibility of the electrode process. When applying the CV method, special attention should be paid to the potential scan window, scan rate, and sweep segments. LSV controls the electrode potential to vary at a constant rate, so the scan rate significantly impacts the results. Compared with other electroanalytical methods, EIS provides more information about the dynamics of electrode process and interface structure. It is not only used to characterize the modification process of electrode but also serves as a signal output for chiral recognition. Generally, the frequency range used for EIS testing should be wide enough and conducted from high to low frequency to obtain sufficient information. DPV and SWV belong to differential pulse voltammetry. SWV effectively reduces the background current caused by double-layer charging current and characterizes the redox process at the interface. It is recognized as the pulse technology with the highest sensitivity and the fastest analysis speed. DPV results in a relatively gentle charging current, thereby reducing the matrix influence from a complex system. DPV shows high Faraday current efficiency, high sensitivity and resolution, and is the preferred electroanalytical technology for electrochemical chiral sensors based on biomaterials. To ensure optimal performance when applying SWV and DPV, it is necessary to adjust the applied pulse amplitude, pulse width, pulse period, and sampling width according to the detected objects and testing environments.

Biomolecules are naturally occurring, optically pure chiral compounds that exhibit excellent biocompatibility, modifiability, low cost, and easy availability. These properties make them widely applicable in the fabrication of electrochemical chiral sensors for the identification of enantiomers. To further improve the chiral recognition efficiency of such sensors, researchers have focused on integrating biomolecules with diverse materials to construct chiral sensing interfaces with enhanced chiral recognition. However, the configuration complexity of biomacromolecules has led to differences in their electrochemical chiral recognition capabilities, which remain poorly understood. Currently, contact angle experiments and ultraviolet-visible spectroscopy (for binding thermodynamics research) are applied to demonstrate the variations in the binding affinity between polysaccharides and enantiomers [146,147,148]. The spatial steric hindrance prevents many recognition sites of proteins from being close to the target substances. Therefore, the interaction between proteins and enantiomers has been simulated through using some solvent-accessible fragments [62,65]. Moreover, there are relatively few reports related to electrochemical chiral recognition based on enzymes and nucleic acids. The relationship between the configuration characteristics of enzymes and nucleic acids and their enantiomer recognition efficiency has been scarcely investigated.

Although significant progress has been made in the development of electrochemical chiral sensing platforms based on biomaterials, which hold great promise for advancing chiral recognition, the development of practical and convenient chiral sensors still faces substantial challenges while offering vast prospects. (i) The configuration complexity of biomacromolecules poses challenges in fully understanding their interaction with chiral molecules. Further elucidation of precise molecular interaction models is essential to enhance the performance of recognition systems based on such interactions. (ii) Most analytical methods are currently capable of determining only the relative enantiomeric composition in racemic mixtures, rather than direct, selective, and quantitative detection of specific enantiomers in complex systems. It should be emphasized that real samples often contain multiple interfering substances, and different enantiomers may coexist within the same system, which greatly restricts the practical applicability of existing techniques. Therefore, it is necessary to construct electrochemical chiral sensors with higher selectivity and sensitivity for practical applications in complex and clinical samples. (iii) Researchers also need to constantly compound various materials and biomolecules to develop electrochemical chiral sensing devices with high recognition performance, thereby meeting the demands of large-scale separation and detection in the practical production of chiral drugs. (iv) Nucleic acid nanotechnology is expected to be further applied in electrochemical chiral recognition. By designing different DNA structures and using a nucleic acid amplification technique [149], the electrical signal generated by the interaction between chiral selectors and enantiomers can be effectively amplified. To simplify the DNA design process, the development of more automated DNA sequence design tools is anticipated. (v) To optimize the chiral recognition interface design of biomaterials, computational chemistry (such as molecular simulation and machine learning) [150,151,152,153] can be employed to predict the binding differences between chiral selectors and enantiomers, thereby accelerating the development of high-performance electrochemical chiral sensors. Moreover, the identification of more suitable chiral selectors represents a highly effective strategy.

## 5. Conclusions

In summary, this review provides an overview of the contributions made by researchers in the field of electrochemical chiral sensing over the past five years, focusing on electrochemical chiral recognition based on biomaterials. (i) Based on a three-point interaction model, the different mechanisms of electrochemical chiral recognition are summarized. The key to chiral recognition lies in the interaction between the chiral selector and the enantiomers, which forms diastereomeric complexes with different stabilities. (ii) The interaction between the chiral selector and the enantiomers causes changes in the electrochemical signals of the system. The application characteristics of different electrochemical analysis techniques (CV, DPV, SWV, and EIS) in chiral recognition are analyzed. (iii) Amino acids and their derivatives, polysaccharides and their derivatives, proteins, nucleic acids, and enzymes can offer chiral recognition sites for the construction of electrochemical chiral sensors. The chiral recognition ability of composite materials formed by these substances and different materials is explored.

The analytical performance of different biomaterials in the field of electrochemical chiral sensing is summarized (Table 1). It has been 19 years since the first biomaterial-based electrochemical chiral sensor was reported. Since then, the sensitivity and interference-resistance of such sensors have been improved through the continuous optimization of composite materials. Different biomaterials exhibit distinct application characteristics (Table 2) and give full play to their advantages to obtain efficient chiral recognition abilities. The effective strategies for improving the efficiency of chiral recognition include biomaterial modification and the formation of composites with other materials (carbon-based materials, MOFs, MNPs). Among these, the integration of MOFs and biomaterials has become a research hotspot. The DPV method provides higher sensitivity and faster analysis time, while significantly reducing the charging current, making it widely applied in electrochemical chiral sensing. In practical applications (such as in human serum and urine, etc.), electrochemical sensors based on biomaterials have been proven to be highly useful because biomaterials offer satisfactory biocompatibility compared with other chiral selectors and are easy to modify. Although electrochemical chiral recognition based on biomaterials has made great progress, several active research directions remain. Notably, there is a lack of specific research on the interaction between the biomaterial conformation and enantiomers. In particular, the relationship between the conformation of biomacromolecules and recognition capability in electrochemical sensors that utilize biomacromolecules as chiral selectors remains unelucidated. At the same time, few of the reported sensors have been commercialized, highlighting the need for continuous efforts to develop sensor devices for chiral separation and recognition of drugs in industrial production. In the future, biomaterial-based electrochemical chiral sensors are expected to gain increasing attention and find broader applications in diverse fields, such as pharmaceutical development, clinical diagnostics, and environmental monitoring.

## Figures and Tables

**Table 1:** Electrochemical chiral sensors based on biomaterials for the recognition of enantiomers.

Biomaterial	Modifier	Method	Mechanism	Analyte	Recognition Ability	LOD	Real Sample	Ref.
Amino acids and their derivatives	L-GO D-GO	EIS and LSV	Three-point interaction	L-Trp D-Trp	I_L_/I_D_ = 1.4, ΔE_P_ = 10 mV I_D_/I_L_ = 1.53, ΔE_P_ = 22 mV	–	–	[34]
	RGO-Au/L-Glu RGO-Au/D-Glu	DPV	Three-point interaction	L-Trp D-Trp	I_L_/I_D_ = 2.56 I_L_/I_D_ = 2.05	0.28 mM 0.86 mM	–	[35]
	L-NGQDs D-NGQDs	EIS and LSV	Three-point interaction	L-Tart D-Tart	–	–	–	[36]
	L-CQDs/TCPP D-CQDs/TCPP	DPV	Three-point interaction	L-Phe D-Phe	I_L_/I_D_ = 2.3 I_D_/I_L_ = 2.7	–	Human serum	[37]
	KB/D-His-ZIF-8	DPV	Three-point interaction	L-Trp D-Trp	I_D_/I_L_ = 5.69	0.51 μM 0.23 μM	Human serum and human urine	[32]
	D-His-ZIF-8@Au@ZIF-8	DPV	Three-point interaction	L-Phe D-Phe	I_L_/I_D_ = 1.97	0.195 mM 0.195 mM 0.588 mM	Human serum	[38]
	D-His-ZIF-8@CoFe-PDA	DPV	Three-point interaction	L-Trp D-Trp	I_L_/I_D_ = 2.01	0.066 mM 0.15 mM	Human serum and human urine	[39]
	L-PCN-224 D-PCN-224	DPV	Three-point interaction	L-Trp D-Trp	I_D_/I_L_ = 2.723 I_L_/I_D_ = 2.682	–	–	[40]
	MOF-Glu MOF-His MOF-Lys	DPV	Three-point interaction	L-Trp, D-Trp L-Phe, D-Phe L-Glu, D-Glu	I_L_/I_D_ = 2.69 I_L_/I_D_ = 2.58 I_L_/I_D_ = 2.00	–	–	[41]
	h-HDGA@ZIF-67	CV	Three-point interaction	L-Pen D-Pen	ΔE_P_ = 272 mV	0.015 μM 0.022 μM	–	[42]
	LCMS DCMS	DPV	Molecular imprinting recognition	L-Trp D-Trp	I_L_/I_D_ = 2.788 I_D_/I_L_ = 3.186	0.012 μM 0.009 μM	Human serum and human urine	[43]
	Au@p-L-cysteine	DPV	Chiral ligand exchange recognition	L-Trp D-Trp	ΔI_D_ = I_blank_ − I_D_ > 0 ΔI_L_ = I_blank_ − I_L_ < 0	75 nM	Human serum	[44]
	Au-D-Met/CS	DPV	Three-point interaction	L-Trp D-Trp	I_L_/I_D_ = 1.54	42.16 pM 7.75 pM	Nutritional supplement	[45]
	L-Cys-AuNPs D-Cys-AuNPs	DPV and EIS	Three-point interaction	R(+)-PRNL S(-)-PRNL	ΔE_P_ = 40 mV	–	–	[33]
	L-His-ZIF	EIS	Three-point interaction	L-Glu	–	0.06 nM	Soy sauce	[46]
Polysaccharides and their derivatives	Cu_2_-β-CD/NH_2_-CS-MWCNTs	DPV	Host–guest recognition	L-Trp D-Trp	I_L_/I_D_ = 1.64 ΔE_P_ = 40 mV	13.4 μM 18.5 μM	Rat serum	[47]
	(CS/PAA)n@PEDOT:PSS	DPV	Three-point interaction	L-Trp D-Trp	I_D_/I_L_ = 6.71 ΔE_P_ = 64 mV	0.33 μM 0.67 μM	–	[48]
	MoS_2_-IL/CS	DPV	Three-point interaction	L-Trp D-Trp	ΔE_P_ = 53.3 mV	–	–	[49]
	RGO/CS	CV and DPV	Three-point interaction	L-Tyr D-Tyr	I_D_/I_L_ = 1.39 ΔI = 5.0 μA	–	–	[50]
	CMC-MIL-88(Fe)	DPV	Three-point interaction	L-Trp D-Trp	I_L_/I_D_ = 2.04	–	Human serum and human urine	[51]
	rGO-PhenCu-CMC	DPV	Chiral ligand exchange recognition	L-Trp D-Trp	I_L_/I_D_ = 2.41	–	–	[52]
	rGO-PANI/CD-Cu-CMC	DPV	–	L-Trp D-Trp	I_L_/I_D_ =3.58	–	–	[53]
	CNT/PANI/SA	DPV	Three-point interaction	L-Trp D-Trp	I_D_/I_L_ = 2.1	–	Human serum and human urine	[54]
	CS-SA	SWV	Three-point interaction	L-Tyr D-Tyr	I_L_/I_D_ = 1.63	29 nM 107 nM	–	[55]
	SA-CS-NGC	DPV	Three-point interaction	L-Trp D-Trp	I_L_/I_D_ = 4.52	–	Human serum and human urine	[56]
	MWCNTs/CMC-CD-Cu	DPV	Chiral ligand exchange recognition	L-Trp D-Trp	I_L_/I_D_ = 2.2	0.81 μM 1.9 μM	Human urine	[57]
	β-CD/CNTs@rGO	DPV	Host–guest recognition	L-Phe D-Phe	–	0.08 μM	–	[58]
	SA/CuNPs/rGO	CV and DPV	Three-point interaction	L-Trp D-Trp	I_L_/I_D_ = 2.11 ΔE_P_ = 36 mV	0.205 μM 0.319 μM	Cow milk, goat milk, beef, and millet	[59]
	GO-CLMOF	DPV	Host–guest recognition	L-MA D-MA	I_L_/I_D_ = 1.8 ΔE_P_ = 40 mV	0.09 mM 0.15 mM	Human urine	[60]
	SGO-NH_2_-βCD/BPNs	SWV	Host–guest recognition	L-Tyr D-Tyr	I_D_/I_L_ = 1.94 ΔI = 0.89 μA	1.74 μM 1.02 μM	–	[61]
Proteins	BSA-GQD	DPV	Biological macromolecule recognition	L-Trp D-Trp	I_L_/I_D_ = 3.67 ΔE_P_ = 96 mV	–	–	[62]
	BSA/MB/MWCNT	DPV	Biological macromolecule recognition	Myo-Ins D-chiro-Ins	–	0.5 μM 1 μM	Commercial pharmaceutical preparation	[63]
	Fe_3_O_4_@COF@BSA	LSV	Biological macromolecule recognition	L-Trp D-Trp	I_L_/I_D_ = 1.45 ΔE_P_ = 23 mV	–	–	[64]
	BSA/TiO_2_	EIS	Biological macromolecule recognition	L-Asp D-Asp	ΔR = 5584.3 Ω	9.39 nM 8.34 nM	–	[65]
	MIPs/HSA	DPV	Molecular imprinting recognition	S-FLX	–	6.43 × 10^−17^ M	–	[66]
	PEI/D-BGAc	DPV	Three-point interaction	L-Trp D-Trp	I_D_/I_L_ = 3.4 ΔE_P_ = 8 mV	0.67 μM 0.33 μM	–	[67]
	α-CD/GSH	DPV	Three-point interaction	L-Trp D-Trp	I_L_/I_D_ = 3.88	–	–	[68]
	GSH-Cu/Pt	DPV	Three-point interaction	L-Tyr D-Tyr	I_L_/I_D_ = 5.11 ΔE_P_ = 104 mV	–	–	[69]
	APS-DPAN-BSA	DPV	Biological macromolecule recognition	L-Trp D-Trp	I_D_/I_L_ = 1.95	0.071 mM 0.0478 mM	–	[70]
Enzymes	β-D-GOD@CS-CNTs	CV	Biological macromolecule recognition	L-Glc D-Glc	–	0.085 mM	Human urine	[71]
	Fe_3_O_4_@Au@Ag@Cu_x_O NPs	DPV	Biological macromolecule recognition	D-Ala	–	52 pM	Human urine	[72]
	DAAO/CNTs	CV	Biological macromolecule recognition	D-Ala	–	7.91 μM	Milk and human urine	[73]
	3D-nCE	EIS	Biological macromolecule recognition	L-Ala	–	10 × 10^−15^ M	-	[74]
Nucleic acids	MIP/dsDNA	DPV	Molecular imprinting recognition	L-Pen	–	2.48 × 10^−16^ M	Penicillamine tablets	[75]
	MIPs/dsDNA	DPV	Molecular imprinting recognition	D-carnitine	–	2.24 × 10^−16^ M	Baby milk powder and weight loss capsules	[76]
	L-Apt@AuNCs D-Apt@AuNCs	DPV	Biological macromolecule recognition	L-Tym D-Tym	I_D_/I_L_ = 4.0 I_L_/I_D_ = 3.0	–	–	[77]
	DNA/CS	DPV	Biological macromolecule recognition	L-Trp D-Trp	I_D_/I_L_ = 4.02	1.67 μM 1.33 μM	–	[78]
	DNA_3/4_/Cu^2+^/MCH/DNA_1/2_/Au	DPV	Biological macromolecule recognition	L-carnitine D-carnitine	–	–	–	[79]
	MCH/HP/Au/Fe_3_O_4_@rGO	DPV	Biological macromolecule recognition	L-His D-His	I_L_/I_D_ = 2.61 I_L_/I_D_ = 2.68	0.28 pM	Human serum	[80]

Limits of detection (LOD): acceptable (>10 μM), low (0.01–10 μM), and considerably low (<0.01 μM); Abbreviations: EIS, electrochemical impedance spectroscopy; LSV, linear sweep voltammetry; DPV, differential pulse voltammetry; CV, cyclic voltammetry; SWV, square wave voltammetry; ΔI, the difference in peak current; ΔE_p_, the difference in peak potential; ΔR, the difference in impedance; I_L_, the peak current of the L isomer; I_D_, the peak current of the D isomer; I_blank_, the peak current of the blank sample; Trp, tryptophan; Tart, tartaric acid; Phe, phenylalanine; Pen, penicillamine; PRNL, propranolol; Glu, glutamic acid; Tyr, tyrosine; MA, mandelic acid; Myo-Ins, myo-inositol; D-chiro-Ins, D-chiro-inositol; Asp, aspartic acid; S-FLX, S-fluoxetine; Glc, glucose; Ala, alanine; Tym, tyrosinamide; His, histidine; Cys, cysteine; Lys, lysine; GO, graphene oxide; RGO/rGO, reduced graphene oxide; NGQDs, nitrogen-doped graphene quantum dots; CQDs, carbon quantum dots; TCPP, porphyrin; KB, ketjen black; ZIF, zeolite imidazole framework; CoFe-PDA, Co^2+^ and Fe^2+^-coordinated polydopamine; PCN-224, metal–organic framework composed of porphyrin ligands and zirconium clusters; MOF, metal–organic framework; h-HDGA, L-Glu terminated bolaamphiphile; LCMS, L-tryptophan imprinted gold nanoparticle surface and the template molecules are removed; DCMS, D-tryptophan imprinted gold nanoparticle surface and the template molecules are removed; p-L-cysteine, poly-L-cysteine; Met, methionine; CS, chitosan; Cu_2_-β-CD, Cu^2+^-modified β-cyclodextrin; MWCNTs/MWCNT, multi-walled carbon nanotubes; PAA, polyacrylic acid; PEDOT:PSS, poly (3,4-ethylenedioxythiophene): poly (styrenesulfonate); MoS_2_-IL, molybdenum disulfide-ionic liquid; CMC, sodium carboxymethyl cellulose; MIL-88(Fe), metal–organic framework prepared using ferric chloride hexahydrate and diaminodiphenylmethane; PhenCu, phenanthroline copper; PANI, polyaniline; CNT/CNTs, carbon nanotubes; SA, sodium alginate; NGC, 3D nitrogen-doped reduced graphene oxide-carbon nanotubes; CLMOF, chiral cross-linked metal–organic framework; SGO, single-layer graphene oxide; βCD/β-CD, β-cyclodextrin; BPNs, black phosphorus nanosheets; BSA, bovine serum albumin; GQD, graphene quantum dots; MB, methylene blue; COF, covalent organic framework; MIPs/MIP, molecularly imprinted polymers; HSA, human serum albumin; PEI, polyethyleneimine; D-BGAc, chiral peptide; α-CD, α-cyclodextrin; GSH, glutathione; Cu/Pt, copper nanoparticles/platinum nanoparticles; APS-DPANI, ammonium persulfate-doped polyaniline; β-D-GOD, β-D-glucose oxidase; DAAO, D-amino acid oxidase; 3D-nCE, 3D-printed nanocomposite carbon electrodes; dsDNA, double-stranded DNA; Apt, aptamer; AuNCs, gold nanoclusters; and MCH, 6-mercaptohexanol. “–” means not provided.

**Table 2:** Summary of different biomaterials used for electrochemical chiral recognition.

Biomaterials	Advantages	Disadvantages	Detection Objects	Lowest LOD	Highest Recognition Efficiency
Amino acids	Clear structures, appropriate molecular sizes, and easy to form complexes	Provides fewer chiral sites and chiral recognition ability is weak	Trp, Tart, Phe, Glu, Pen, and PRNL	42.16 pM (L-Trp) 7.75 pM (D-Trp) [45]	I_D-Trp_/I_L-Trp_ = 5.69 [38]
Polysaccharides	Good biocompatibility, low toxicity, good hydrophilicity, and easy modification	Easy agglomeration and poor conductivity	Trp, Tyr, Phe, and MA	29 nM (L-Tyr) 107 nM (D-Tyr) [55]	I_D-Trp_/I_L-Trp_ = 6.71 [48]
Proteins	Rich functional groups, easily accessible, and good solubility	Adsorption occurs on the electrode surface and covers the redox sites	Trp, Inositol, Asp, FLX, and Tyr	6.43 × 10^−17^ M (S-FLX) [66]	I_L-Tyr_/I_D-Tyr_ = 5.11 [69]
Enzymes	High specificity, sensitivity, and interference-resistance ability	Easily affected by pH, temperature, and solvent	Glc and Ala	10 × 10^−15^ M (L-Ala) [74]	–
Nucleic acid	Biocompatibility, controllable self-assembly, and stable chemical properties	Limited recognition ability for natural DNA and uncertainty of design	Pen, carnitine, Tym, Trp, and His	2.24 × 10^−15^ M (D-carnitine) [76]	I_D-Trp_/I_L-Trp_ = 4.02 [78]

Abbreviations: I_L-Trp_, the peak current of L-Trp; I_D-Trp_, the peak current of D-Trp; I_L-Tyr_, the peak current of L-Tyr; I_D-Tyr_, the peak current of D-Tyr; Trp, tryptophan; Tart, tartaric acid; Phe, phenylalanine; Glu, glutamic acid; Pen, penicillamine; PRNL, propranolol; Tyr, tyrosine; MA, mandelic acid; Asp, aspartic acid; FLX, fluoxetine; Glc, glucose; Ala, alanine; Tym, tyrosinamide; His, histidine. “–” means not provided.

## Data Availability

Not applicable.
